# The Effects of Dexmedetomidine on Children Undergoing Magnetic Resonance Imaging: A Systematic Review and Meta-Analysis

**DOI:** 10.3390/children10060948

**Published:** 2023-05-26

**Authors:** Valentina-Anastasia Angelopoulou, Abraham Pouliakis, Nikolaos Alexiou, Parthena Ioannidi, Dimitra Vagiona, Konstantinos Ekmektzoglou, Theodoros Xanthos, Theodora Boutsikou, Zoi Iliodromiti, Nikoletta Iacovidou

**Affiliations:** 1 Department of Radiology, General Hospital of Elefsina “Thriasio”, 19600 Attica, Greece; valeange274@gmail.com; 2 Postgraduate Study Program (MSc) “Resuscitation”, School of Medicine, National and Kapodistrian University of Athens, 11527 Athens, Greece; ekmektzo@yahoo.gr (K.E.);; 3 Second Department of Pathology, “Attikon” University Hospital, National and Kapodistrian University of Athens, 12464 Athens, Greece; 4 First Department of Internal Medicine, General Hospital of Elefsina “Thriasio”, 19600 Attica, Greece; nicktavros@yahoo.gr; 5 Department of Invasive Radiology, General Hospital of Athens “Evangelismos”, 10676 Athens, Greece; drpopy@yahoo.com; 6 European Board of Interventional Radiology (EBIR), 1010 Vienna, Austria; 7 Primary Health Center of Nevrokopi, General Hospital of Drama, 66100 Drama, Greece; dimvagio@yahoo.gr; 8 School of Medicine, European University Cyprus, 2404 Nicosia, Cyprus; 9 School of Health Sciences, University of West Attica, 12243 Athens, Greece; theodorosxanthos@yahoo.com; 10 Department of Neonatology, “Aretaieio” Hospital, School of Medicine, National and Kapodistrian University of Athens, 11528 Athens, Greece; theobtsk@gmail.com (T.B.); ziliodromiti@yahoo.gr (Z.I.)

**Keywords:** dexmedetomidine, pediatric sedation, magnetic resonance tomography, anesthetics, effectiveness, adverse effects, safety

## Abstract

Background: Magnetic Resonance Imaging (MRI) is a valuable diagnostic tool but often requires sedation to complete, especially in children. Dexmedetomidine (DEX) is an a2 agonist, for which there are experimental findings that support its potential neuroprotective effects. Given the potential risks of anesthetic drugs, we ran this study to examine DEX’s effectiveness and cardiopulmonary safety as a sedative drug for children undergoing MRI. Material and Methods: Systematic research was conducted in PubMed, Google Scholar, Scopus and Cochrane databases for randomized controlled trials published between 2010 and 6th/2022 and involving children undergoing MRI who received DEX as sedative medication. The records which met the including criteria, after indexing via the PRISMA chart and assessing for bias, were processed, and a meta-analysis was carried out with the random effects method. Results: Thirteen studies were included. Out of 6204 measurements obtained, in 4626, it was planned for the participants to only receive DEX (measure group) as an anesthetic drug throughout the procedure. The participants’ mean age was 57 months (Ι^2^ = 4%, τ^2^ = 0.5317, *p* = 0.40). A total of 5.6% (95% CI: 0.6–14.1%, I^2^ = 98%, *p* < 0.01) of the patients needed a second dose of DEX. In total, 6% (95% CI: 1–15%, I^2^ = 93%, τ^2^ = 0.0454, *p* < 0.01) required the administration of another drug, besides DEX, to complete the imaging (sedation failure). The effectiveness of the only-DEX method was 99% (95% CI: 97.5–100%, I^2^ = 81%, τ^2^ = 0.0107, *p* < 0.01). The whole rate of adverse events was 15% (95% CI: 9.3–21.5%, I^2^ = 92%, *p* < 0.01). Hypotension was reported in 8.7% of the cases (95% CI: 3.1–16.4%, I^2^ = 84%, *p* < 0.01), hypertension in 1.1% (95% CI: 0–5.4%, I^2^ = 89%, *p* < 0.01), bradycardia in 10% (95% CI: 4–18%, I^2^ = 95%, *p* < 0.01) and desaturation in 1.2% (95% CI: 0–4%, I^2^ = 68%, *p* < 0.01). There was no statistically significant incidence in respiratory rate decrease (comparing the children who received DEX to their baseline). Five cases of vomiting and one of apnea were recorded. Conclusions: Given that DEX seems to be an effective as well as respiratory and hemodynamically safe drug, it may be a future spotlight in (pediatric) sedation for imaging procedures such as MRI.

## 1. Introduction

Magnetic Resonance Imaging (MRI) is very important for early, targeted, effective and valid diagnoses and therefore the early initiation of the treatment of many diseases; however, the administration of sedative drugs, with propofol, chloral hydrate, midazolam and ketamine being amongst the most common sedative drugs used [1,2,3,4,5,6,7,8,9], is often required for MRI to be completed, especially in the pediatric population. Nevertheless, currently available anesthetic drugs’ possible side effects, such as respiratory and/or cardiovascular depression, addiction, psychotic disorders, mutagenic effects and reductions in neurons in the limbic system, with the latter possibly leading to permanent neurological disorders, pose several limitations regarding their use [10,11,12,13,14,15,16] and require the careful evaluation of their safety profile to select the appropriate method to monitor patients during and after the sedation procedure. Therefore, the need for research into new drugs with better safety profiles has arisen. Dexmedetomidine (DEX), a selective Alpha-2 agonist, is a relatively new drug, with characteristics such as better respiratory tolerance, a safer cardiovascular profile, the ability to cause loss of consciousness similar to normal sleep and even potential neuroprotective effects, which make it an attractive sedativeagent [7,17,18,19]. Dexmedetomidine is a suitable alternative to halogenated general anesthesia sedation options for pediatric MRI, aiming to reduce the level of exposure to conventional anesthetic agents and invasive ventilation [20]. However, until now, research has focused on animal experimental stages or clinical studies comparing and/or combining DEX to and with other sedative drugs in children undergoing MRI. We conducted this meta-analysis with the purpose of examining and collecting data about DEX’s effectiveness (referring to those who successfully completed the process) and safety profile (i.e., the rate and specification of adverse effects), when administered as a solo-sedative drug in the aforementioned population.

## 2. Materials and Methods

We performed a systematic review for randomized controlled trials in human species which were published from 2010 to June 2022 and included pediatric patients (<18 yo) who received dexmedetomidine before MRI examination. This study was performed according to the PRISMA (Preferred Reporting Items for Systematic Reviews and MetaAnalyses) guidelines [21].

### 2.1. Search Strategy

This systematic review was conducted through the PubMed, Google Scholar, Scopus and Cochrane databases of medical literature for human studies. For this purpose, we used the PICO method:

{Component Query: 

P: (Patient, Problem or Population): (((child*[Title/Abstract])OR(kid[Title/Abstract])OR(kids[Title/Abstract]) OR (pediatric[Title/Abstract]) OR (infant*[Title/Abstract])) AND ((MRI[Title/Abstract]) OR (Magnetic*[Title/Abstract])))

I (Intervention): ((dexmedetomidine*[Title/Abstract]) OR (DEX[Title/Abstract]))

C (Comparison, control or comparator): Not used

O (Outcome): ((safe*[Title/Abstract])OR(efficacy*[Title/Abstract])OR(efficient*[Title/Abstract]) OR (effective*[Title/Abstract]) OR (adverse*[Title/Abstract]) OR (complication*[Title/Abstract]))

Τ (Time): Not used

S (Setting): Not used

and thus, the following PICO question arose—PICO Question: (((child*[Title/Abstract])OR(kid[Title/Abstract])OR(kids[Title/Abstract]) OR (pediatric[Title/Abstract]) OR (infant*[Title/Abstract])) AND ((MRI[Title/Abstract]) OR (Magnetic*[Title/Abstract]))) AND ((dexmedetomidine*[Title/Abstract]) OR (DEX[Title/Abstract])) AND ((safe*[Title/Abstract]) OR (efficacy*[Title/Abstract]) OR (efficient*[Title/Abstract]) OR (effective*[Title/Abstract]) OR (adverse*[Title/Abstract]) OR (complication*[Title/Abstract]))

and the equivalent query for the Google Scholar, Scopus or Cochrane databases was:

(((child*) OR (kid) OR (kids) OR (pediatric) OR (infant*)) AND ((MRI) OR (Magnetic*))) AND ((dexmedetomidine*) OR (DEX)) AND ((safe*) OR (efficacy*) OR (efficient*) OR (effective*) OR (adverse*) OR (complication*)).

Additionally, we scrutinized other records with the same selection criteria (see below) and for the same publication period, using as search key words: pediatric sedation, magnetic tomography or MRI and dexmedetomidine. 

Adolescents were not included since they mostly do cooperate during MRI examination and sedation is not often required.

### 2.2. Study Selection and Data Extraction

#### 2.2.1. Selection Criteria

Inclusion and exclusion criteria were defined prior to the literature search. Hence, the following criteria had to be met for studies to be included in this meta-analysis: 

(1) Published as full articles; (2) written in English; and (3) randomized controlled trials with extractable data about DEX as a monotherapy in children undergoing MRI (specifically: participants’ demographic data, doses administered, route of administration, need for more/or other drug(s), successful completion of the process, adverse events after administration and recovery operations). 

#### 2.2.2. Exclusion Criteria Were the Following

(1) Studies without data for retrieval; (2) duplicate studies; and (3) the less informative publication of two on the same study.

Two of the authors (A.V. and I.P.) assessed the title and/or abstract of the identified records as well as the full content in order to clarify if they meet the criteria or not.

#### 2.2.3. Extraction of Data 

Two of the authors (A.V. and E.K.) independently extracted the above data from suitable studies using a standard form. Any disagreement was settled by further discussion, and a third author (A.N.) was consulted. Then, all extracted data were tabulated using Excel spreadsheets.

### 2.3. Quality and Risk of Bias Assessment

Concerning the evaluation of the existence of possible bias risks, all enrolled studies were scrutinized, by two of the authors (A.V. and V.D.), for relevant bias factors, i.e., (a) random sequence generation (selection bias), (b) allocation concealment (selection bias), (c) the blinding of participants and personnel (performance bias), (d) the blinding of outcome assessment (detection bias), (e) incomplete outcome data (attrition bias), (f) selective reporting (reporting bias) and (g) other bias. For this purpose, a revised version of the risk-of-bias tool for randomized trials (RoB-2) was used [22]. The risk of bias was classified as: (i) low, (ii) some concerns and (iii) high. 

### 2.4. Statistical Methods

This meta-analysis was performed using the random effects method. As it was impossible to obtain data for each individual patient, analysis was carried out on the aggregated data, which were extracted from the tables and/or results sections of the included studies and after the determination of their significance. Additionally, in cases with different populations, data were reported for the individual groups. The meta-analysis was conducted using the R statistical computing language (edition 4.0.4.) [23], in the Microsoft Windows environment using the package Meta (version 4.18.0) [24,25]. In each included study, results were presented in a different manner; however, in meta-analyses, the mean values and standard deviation of each variable reported in the studies need to be known. In cases where these were not explicitly reported in the studies, the mean and 1st and 3rd quadrants were used to approach an estimate, as suggested by Hozo et al. [26]. Moreover, if the maximum and minimum values were also available, then an improved method for this estimation (as suggested by Bland [27]) was used. For that purpose, the Deep Meta Tool, Version 1 [28] was used. 

## 3. Results

### 3.1. Study Selection—Characteristics of Included Records

**Figure 1:**
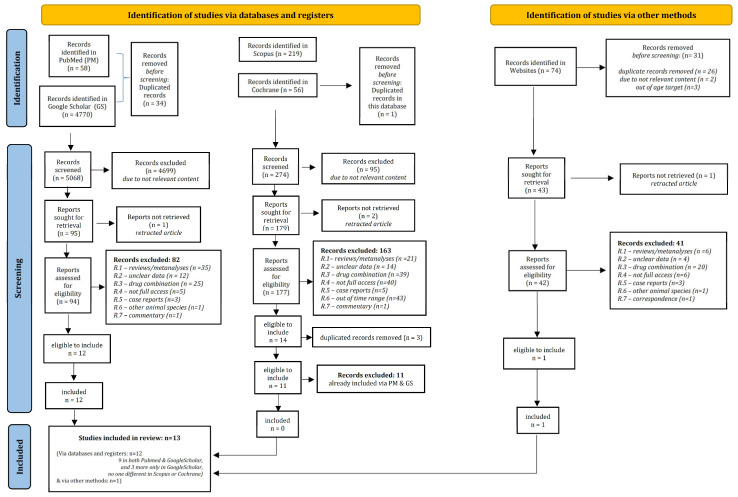
PRISMA chart.

After assessing their title and/or the abstract, as well as the full content, and following the PRISMA flow chart (see Figure 1) [21,29] for indexing them, thirteen records met the including criteria and were utilized in the subsequent analysis [13,30,31,32,33,34,35,36,37,38,39,40,41]. Information about the included records, populations, sub-groups, induction doses and maintenance doses are presented in Table 1.

### 3.2. Quality Assessment—Risk of Bias

After the assessment of risk of bias was completed, a traffic light diagram was produced; in this diagram, the outcomes of the evaluation for each enrolled study and for each of the five different domains are presented (D1: randomization process; D2: deviations from the intended interventions; D3: missing outcome data; D4: measurement of the outcome; D5: selection of the reported result) separately, as well as overall (see Supplementary Figure S1). As has already been reported, the risk of bias was classified as low, some concerns and high. For the first four domains, the risk of bias would be classified as low (low risk from 61.5% up to 100%), while in the fifth domain would be classified as marginal or with some concerns at a rate of 61.5%. There were some concerns regarding the overall bias, as it was about 23.1%, low, or 76.9%, with some concerns (Supplementary Figure S2). There was no study with a high risk of bias, neither at each different domain nor cumulatively.

### 3.3. Analysis’ Results

Participants’ age: This study is about a pediatric population (<18 yo). After removing extreme ages and records with very small numbers of participants, the mean age was 57 months (Ι^2^ = 4%, τ^2^ = 0.5317, *p* = 0.40) (see Supplementary Figure S4), and the relevant publication bias was low (see Supplementary Figure S5).

Induction and maintenance dose of DEX: Detailed information about induction to anesthesia and maintenance doses are presented in Table 1. For these two parameters, due to lack of standard deviation in many of the included studies, the weighted average was calculated. The induction dose was 2.8 ± 0.5 μg/Kg and was administered over 10 min, and the maintenance dose was 1.8 ± 0.4 μg/Kg/h.

Additional dose of DEX: This refers to the administration of more than one bolus doses of DEX, except the induction and maintenance doses. In total, 5.6% (95% CI: 0.6–14.1%) of the patients required an additional dose of DEX (Ι^2^ = 98%, τ^2^ = 0.0455, *p* < 0.01). The weighted mean of the second dose was 2.9 ± 0.4 µg/Kg (see Supplementary Figure S7).

**Figure 2:**
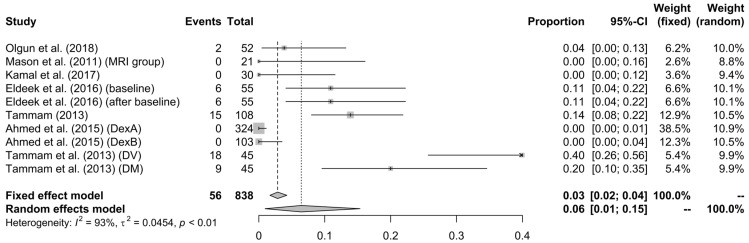
Forest plot of DEX failure [13,31,34,35,36,38,39].

DEX’s failure (use of another, except DEX, drug): In cases in which DEX alone was not adequate enough for the completion of the imaging examination, the administration of another drug was required; this was considered/defined as DEX failure. The percentage of DEX failure reported in the studies fluctuated from 0% to 40%, which meant high heterogeneity; however, in total, 6% (95% CI: 1%–15%, I^2^ = 93%, τ^2^ = 0.0454, *p* < 0.01) needed an additive, except the DEX drug (see Figure 2).

Onset time: This is the time between the administration of DEX until the onset of sedative effects; this time was 12.5 min, while it varied from 7 to 19 min in the included studies (see Supplementary Figure S10). 

Recovery time: In some studies, it was defined as the time between the end of sedation until the patient’s dismissal, while in other studies, as was defined as until the communication level was restored, which contributed to the ambiguity of the result. The mean recovery time was 32 min (range 9–77 min) (see Supplementary Figure S12); however, as expected, the bias was high (see Supplementary Figure S13).

Total time: This refers to the period since the beginning of the administration of sedation until the completion of the imaging examination. The reported range was from 23 to 60 min; the aggregated time was calculated as 39.2 min (95% CI = 36.9–41.6 min, I^2^ = 71%, *p* = 0.02) (see Supplementary Figure S14).

**Figure 3:**
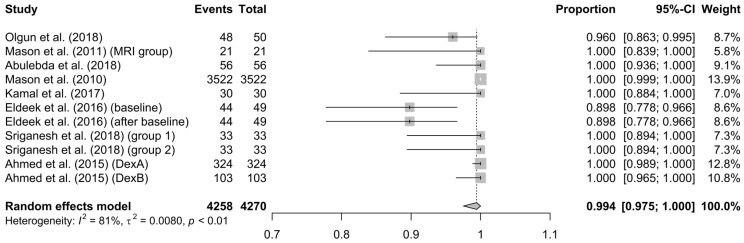
Forest plot about the efficiency of the method [13,31,32,33,34,35,37,38].

Efficacy of only-DEX method: This refers to the percentage of patients who managed to complete the procedure successfully (with a satisfactory image obtained and without interruption of the procedure due to an adverse effect) and received only dexmedetomidine as suppressant medication. The efficiency of the only-DEX method was 99% (95% CI = 97.5–100%), and the heterogeneity of the results was characterized as small (I^2^ = 81%, *p* < 0.01) (see Figure 3), while the bias regarding publication bias was low (see Supplementary Figure S16).

**Figure 4:**
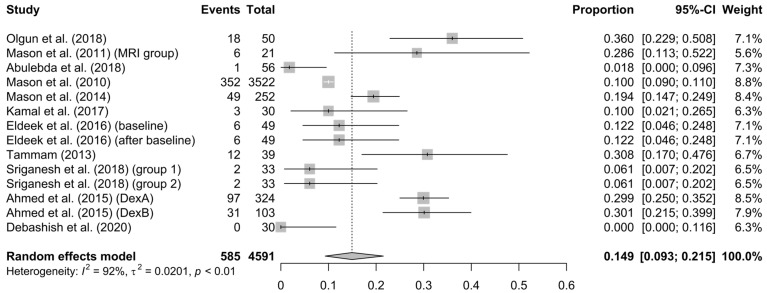
Forest plot of method’s overall safety [13,31,32,33,35,36,37,38,40,41].

Safety of DEX: This is the overall record of the occurrence of adverse effects (such as bradycardia; hypotension; hypertension; respiratory effects—desaturation; apneas—postanesthetic nausea and vomiting; each of them is analyzed below). The rate of side effects in the meta-analysis was determined to be 15% (95% CI = 9.3–21.5%), with high heterogeneity (I^2^ = 92%, *p* < 0.01) (see Figure 4) but low bias (see Supplementary Figure S17).

Hypotension: This was defined as a drop in the mean arterial pressure (MAP) or systolic blood pressure (SBP) more than 20% from the patients’ predicted reference levels and occurred with an overall rate of 8.7% of the cases (95% CI = 3.1%–16.4%, I^2^ = 84%, *p* < 0.01) (see Supplementary Figure S18).

Hypertension: Hypertension was defined as an increase in MAP or SBP greater than 20% of the upper predicted limit. The overall rate was 1.1% (95% CI = 0–5.4%, I^2^ = 89%, *p* < 0.01) (see Supplementary Figure S20).

**Figure 5:**
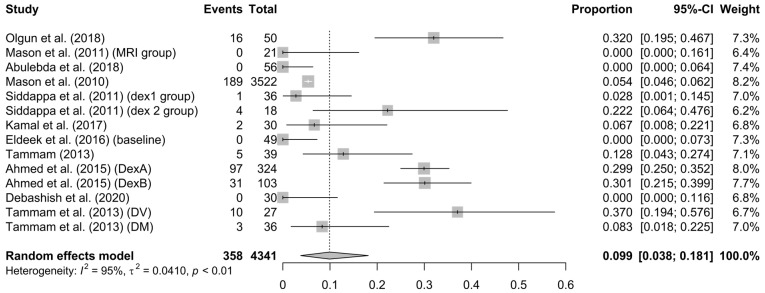
Forest plot for bradycardia [13,30,31,32,33,34,35,36,38,39,40].

Bradycardia: Bradycardia was considered as a drop in heart rate of more than 20% of the predicted reference level or less than 60 beats/min. Bradycardia was reported with an aggregated rate of 10% (95% CI = 4–18%) but with significant heterogeneity (I^2^ = 95%, *p* < 0.01) (see Figure 5).

**Figure 6:**
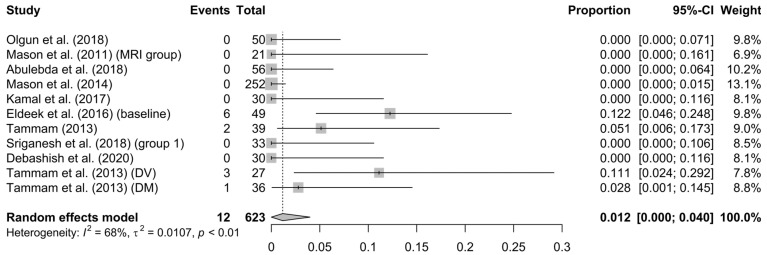
Forest plot of desaturation [13,31,32,34,35,36,37,39,40,41].

Desaturation: Desaturation was determined as a peripheral blood oxygen saturation value (SpO2) below 92–93%. Aggregately, desaturation appeared in just 1.2% of the patients (95% CI = 0–4%), with a relatively low heterogeneity of 68% (*p* < 0.01) (see Figure 6).

Other adverse events that were mentioned in the included studies: There was no statistically significant incidence of decreases in respiratory rate (when comparing the children who received DEX to their baseline) and in total, five isolated cases of vomiting and one case of apnea were recorded.

## 4. Discussion

This study involved a pediatric population (participants’ mean age: 57 months, Ι^2^ = 4%, τ^2^ = 0.5317, *p* = 0.40) undergoing MRI and was carried out in order to assess the efficacy and safety profile of a relatively new drug, i.e., dexmedetomidine. The main reason was to continuously raise awareness about the use of sedative drugs in children, taking into consideration the potential of long-term complications of these (sedative drugs) on children’s developing nervous systems. Until now, there have been data comparing DEX with classical anesthetics, but there are no reports that have collected evidence of the efficacy and safety for the substance under investigation. 

Of the 6204 participants, in 6084, DEX was involved in the medication, and in 4626 of them, DEX was the only anesthetic drug used throughout the procedure. We focused on subgroups that received only DEX as the sedative, in order to ensure the evaluation of the effects of this drug and to limit the possible error in the effects’ interpretation.

After the analysis of the included studies, in 6% (95% CI = 1–15%, I^2^ = 93%, τ^2^ = 0.0454, *p* < 0.01) of the population, DEX was inadequate to fulfill its purpose; therefore, in order to complete the imaging, the administration of another drug was required (DEX failure). This result is conformant to the results of Sriganesh et al. [37] comparing DEX to propofol, i.e., another drug was required to complete the imaging examination in 8.3% and 17%, respectively. However, Tammam et al. [36] showed that when DEX is combined with ketamine, the failure rate decreases to 5.6% (*p* = 0.007) (despite the fact that for each individual drug, the failure rate was 27.8% and 22.2%, respectively). These differences in the agreement of the results of the current study with the two studies mentioned above [36,37] could be interpreted/explained if we consider the different route and time of administration in the two studies (bolus intramuscular [36] vs. continuous intravenous infusion [37]) and the fact that our study reported the overall outcome. However, in the population who received only DEX as a sedative, the efficacy was up to 99% (95% CI: 97.5–100%), while the heterogeneity (Ι^2^ = 81%, τ^2^ = 0.0080, *p* < 0.01) and the bias were both low. As a result, we could say that DEX in an effective sedative drug.

The onset time of DEX’s sedative effects was calculated to be 12.5 min (95% CI: 7–19 min, I^2^ = 99%, *p* < 0.01), which is compatible with the results of other studies [40] but is also significantly longer compared to ketamine’s, i.e., 6.30 ± 1.32 min (*p* = 0.001) [33].

Regarding the issue of DEX’s safety and the side effects, in all studies in which data about adverse events were reported, the cumulative risk for side effects was 15% (95% CI: 9.3–21.5%, I^2^ = 92%, τ^2^ = 0.0201 *p* < 0.01) with low bias. The incidence of hypotension was 8.7% (95% CI: 3.1–16.4%, I^2^ = 84%, τ^2^ = 0.0276, *p* < 0.01). When DEX was compared to propofol, it seemed to be superior, as Abulebda et al. [32] reported the appearance of hypotension in 3.6% and 39% of cases, respectively (*p* < 0.0001). In contrast, when Eldeek et al. [35] compared DEX with ketamine, they observed a statistically significantly (*p* < 0.001) greater decline from baseline during DEX administration. On the other hand, hypertension was recorded in 1.1% (95% CI = 0–5.4%, Ι^2^ = 89%, τ^2^ = 0.0144, *p* < 0.01). Earlier, Masonet et al. [33] observed that hypertension was dependent on the number of repeated doses (OR: 2%, 95% CI: 1.4–2.8), as well as the patient’s age (with 1 year cut-off). Additionally, the incidence of hypertension was lower after DEX administration than after ketamine, as Tammam et al. [36] showed, where the frequency was 0% and 12.96%, respectively. Moreover, regarding cardiovascular effects, bradycardia is one of the expected side effects of DEX (as they are reported in bibliography). In our records, bradycardia was observed in 10% (95% CI = 4–18%, I^2^ = 95%, τ^2^ = 0.0410, *p* < 0.01) of the cases. However, no interventions were required to adjust heart rate. This incidence matches to that of Tammam et al. [36], which was 9.3%. Moreover, when DEX was compared with propofol, in the study by Abulebda et al. [32], it caused statistically significantly (*p* < 0.0001) less bradycardia (3.6% of cases) than propofol (27.7% of cases). In terms of respiratory function, desaturation was observed in 1.2% of cases (95% CI = 0–4%, I^2^ = 68%, τ^2^ = 0.0107, *p* < 0.01). This was also confirmed by Kamal et al. [34], who reported that DEX is superior to propofol, as well as by Tammam et al. [36], who compared DEX and ketamine, and the respective incidence of desaturation was 3.7% and 11.1%, respectively. With regard to the insignificant influence of DEX on the respiratory rate, as was recorded in our study in total, this result is in agreement with previous results, in which DEX seemed to be superior to propofol [34] and ketamine [35]. Additionally, one event of apnea was referred, which was self-restored. Among all records, five isolated events of vomiting were reported. This result agrees with the results of Tammam et al. [36], whereas DEX caused vomiting in 1.85% of patients versus ketamine, which caused it in 14.8%.

However, should be noticed that all the presented outcomes were obtained after the administration of the initial dose of DEX in a period of ten minutes, and after that, the initiation of maintenance infusion began. No results were reported for faster or slower administration. This should be considered in conjunction with the reports of respiratory instability inducted by the rapid injection of DEX (in two minutes) [35].

Our study has limitations. One of them is the significant heterogeneity in some of the results. We could mention the need for a second dose of DEX, which was, in total, needed in 5.6% of cases (95% CI: 0.6–14.1%), but in several studies, this varied from 0% to 100% (Ι^2^: 98%, τ^2^: 0.0455, *p* < 0.01). In addition, the percentage of patients that required another drug, except DEX, fluctuated from 0% to 40% in the included studies, but in total, it was 6% (95% CI = 1–15%, Ι^2^ = 93%, τ^2^ = 0.0454, *p* < 0.01). Moreover, we may refer to the onset time, which was found to be 12.5 min (95% CI: 7–19 min, I^2^ = 99%, *p* < 0.01), as well as to the recovery time, which, due to different definition among the several studies, varied from 9 to 77 min (this was cumulatively estimated at 32 min). The great heterogeneity could have been eliminated if we had only included specific studies in our meta-analysis. However, this would be also a limitation, since by excluding studies from the statistical analysis, much important information about the wider efficacy and actions of the drug would have been rejected. As a conclusion, more targeted studies are required.

## 5. Conclusions

In conclusion, dexmedetomidine as a single sedative, with an induction dose of 2.8 ± 0.5 μg/Kg administered over 10 min and a maintenance dose of 1.8 ± 0.4 μg/Kg/h, had efficacy of 99% (95% CI: 97.5–100%, Ι^2^ = 81%, τ^2^ = 0.0080, *p* < 0.01) with total risk for adverse events of 15% (95% CI: 9.3–21.5%, Ι^2^ = 92%, *p* < 0.01), with none requiring special treatment. In terms of respiratory functions, DEX was better tolerated than classic sedative medications. Concerning the cardiovascular system, the risk for bradycardia was 10%, and the risk for arterial blood pressure effects was 8.7% and 1.1% for hypotension and hypertension, respectively. Given the fact that some of the reported results have significant heterogeneity, more focused research is needed in order to clarify any remaining questions, especially before using dexmedetomidine in vulnerable populations such as children.

## Figures and Tables

**Table 1:** Information about the included records, populations, sub-groups, induction doses and maintenance doses.

Ref.	Authors	Publication Year	Type of Study	N	Sub-Groups	N1	D	D1	Dose D1 (μg/kg)	Route D1	Maintenance (μg/kg/h)
[13]	Olgun et al.	2018	retrospective chart review	52	_	52	50	49	4	IN	ΝR
[30]	Siddappa et al.	2011	retrospective clinical chart review	76	dex1		36	36	2	IV	1
					dex2		18	0	2	IV	1
[31]	Mason et al.	2011	retrospective review of clinical data	65	ΜRI	21	21	20	2.8 ± 0.6	IM	NR
[32]	Abulebda et al.	2018	retrospective chart review	56	_	56	56		3.41 ± 1.04	IV	NR
[33]	Mason et al.	2010	RCT—retrospective review of institute data-base	3522	total	3522	3522	2645	3	IV	2
[34]	Kamal et al.	2017	prospective randomized study	30	_	30	30		2	IV	1–1.5
[35]	Eldeek et al.	2016	randomized prospective comparative study	55	_	55	49	49	1	IV	0.5–0.75
[36]	Tammam	2013	prospective double-blind comparative study	108		108	39	39	3	IM	NR
[37]	Sriganesh et al.	2018	prospective randomized	36	_	36	33	33	2	IV	2
[38]	Ahmed et al.	2015	retrospective institutional review	427	dexA	324	324	324	2	IV	1
					dexB	103	103	0	2	IV	1
[39]	Tammam et al.	2013	double-blind, comparative, randomized study	90	DV	45	27	45	1	IV	1
					DM	45	36	45	3	IM	_
[40]	Debashish et al.	2020	cross-sectional study	30	_	30	30	30	1	IV	0.2–0.7
[41]	Mason et al.	2014	retrospective institute chart review	1657	3 (1 into consideration)	1657	252	140	3	IV	2

Abbreviations: N: population of patients received DEX, dex1 and dexA: subgroups who received only one dose of DEX, dex2 and dexB: subgroups who received more than a single dose of DEX, MRI: subgroup for magnetic tomography (the other subgroups of that study underwent other imaging techniques), DV: subgroup who received DEX intravascular, DM: subgroup who received DEX intramuscular, N1: total cases with DEX, D: patients received ONLY-DEX. D1: patients had only 1 dose of DEX, Route D1: route of 1st dose’s administration, IN: intranasal, IV: intravascular, IM: intramuscular, NR: not reported.

## Data Availability

Data is available from the relevant publications.

## Supplementary Materials

The following supporting information can be downloaded at: https://www.mdpi.com/article/10.3390/children10060948/s1, File with meta-analysis Figures S1–S23, Figure S1: bias assessment diagram formed after evaluating the studies with RoB-2 tool, Figure S2: bias assessment as percentage, after evaluating the studies with RoB-2 tool, Figure S3: bias assessment as percentage, after evaluating the studies with RoB-2 tool, Figure S4: Forest plot of participants’ age (months), Figure S5: Funnel plot of participants’ age (months), Figure S6: Funnel plot about the induction dose of DEX, Figure S7: Forest plot about the need of additional dose of DEX, Figure S8: Funnel plot about additional doses of DEX, Figure S9: Funnel plot about DEX’s failure, Figure S10: Forest plot of onset time, Figure S11: Funnel plot of onset time, Figure S12: Forest plot for the recovery time, Figure S13: Funnel plot about recovery time, Figure S14: Forest plot of total time, Figure S15: Funnel plot of total time, Figure S16: Funnel plot of method’s efficiency, Figure S17: Funnel plot of method’s safety, Figure S18: Forest plot of hypotension, Figure S19: Funnel plot for hypotension, Figure S20: Forest plot of hypertension, Figure S21: Funnel plot for hypertension, Figure S22: Funnel plot for bradycardia, Figure S23: Funnel plot for desaturation).
