# Supplementary material for: The Effects of Dexmedetomidine on Children Undergoing Magnetic Resonance Imaging: A Systematic Review and Meta-Analysis
Source: Children (Basel). 2023 May 26;10(6):948. doi: 10.3390/children10060948 (PMC10297262; doi:10.3390/children10060948)
Supplement: Supplementary file 1 [file children-10-00948-s001.zip › S1 Angelopoulou V.A. - DEX.MRI - supplementary figures with chages.pdf]

THE EFFECTS OF DEXMEDETOMIDINE ON CHILDREN UNDERGOING MAGNETIC RESONANCE IMAGING: A SYSTEMATIC REVIEW & ME-TA-ANALYSIS.

Angelopoulou Valentina-Anastasia[1,2,*], Pouliakis Abraham[3,*], Alexiou Nikolaos[4], Ioannidi Parthena[5,6], Vagiona Dimitra[7], Ekmektzoglou Konstantinos[2,8] , Xanthos Theodoros[9], Boutsikou Theodora[10], Iliodromiti Zoi[10] and Iacovidou Nikoletta[2,10]

1 Department of Radiology, General Hospital of Elefsina "Thriasio", Attica, Greece
2 Postgraduate Study Program (MSc) "Resuscitation", School of Medicine, National and Kapodistrian University of Athens, Greece
3 Second Department of Pathology, "Attikon" University Hospital, National and Kapodistrian University of Athens, Athens, Greece
4 First Department of Internal Medicine, General Hospital of Elefsina "Thriasio", Attica, Greece
5 Department of Invasive Radiology, General Hospital of Athens "Evangelismos", Athens, Greece
6 European Board of Interventional Radiology (EBIR)
7 Primary Health Center of Nevrokopi, General Hospital of Drama, Drama, Greece
8 School of Medicine, European University Cyprus, Nicosia, Cyprus
9 School of Health Sciences, University of West Attica, Athens, Greece
10 Department of Neonatology, "Aretaieio" Hospital, School of Medine, National and Kapodistrian Uni-versity of Athens, Athens, Greece

***Correspondence:**
Abraham Pouliakis:
apou1967@gmail.com; phone number: mob: +30 697 2846977; tel: +30 210 5831958 & 5831953; fax: +30 210 5831942
Angelopoulou Valentina-Anastasia:
valeange274@gmail.com; phone number: mob: +30 697 8142824

FIGURES of BIAS ASSESSMENT of the included studies

Unique ID	Study ID	Experimental	Comparator	Outcome	Weight	D1	D2	D3	D4	D5	Overall
1	Olgun et al (2018)	NA	NA	NA	1	!	+	+	+	+	!
2	Siddappa et al (2011)	NA	NA	NA	1	+	!	+	+	!	!
3	Mason et al (2011)	NA	NA	NA	1	!	!	+	+	+	!
4	Abulebda et al (2018)	NA	NA	NA	1	+	+	+	+	+	+
5	Mason et al (2010)	NA	NA	NA	1	!	+	+	+	!	!
6	Kamal et al (2017)	NA	NA	NA	1	+	+	+	+	!	!
7	Eldeek et al (2016)	NA	NA	NA	1	+	+	+	+	!	!
8	Tammam (2013)	NA	NA	NA	1	+	+	+	+	!	!
9	Sriganesh et al (2018)	NA	NA	NA	1	+	+	+	+	+	+
10	Ahmed et al (2015)	NA	NA	NA	1	!	+	+	+	!	!
11	Tammam et al (2013)	NA	NA	NA	1	+	+	+	+	!	!
12	Debashish et al (2020)	NA	NA	NA	1	+	+	+	+	+	+
13	Mason et al (2014)	NA	NA	NA	1	!	+	+	+	!	!

+	Low risk
!	Some concerns
-	High risk
D1	Randomisation process
D2	Deviations from the intended interventions
D3	Missing outcome data
D4	Measurement of the outcome
D5	Selection of the reported result

Sup.fig.1: Bias assessment diagram formed after evaluating the studies with RoB-2 tool

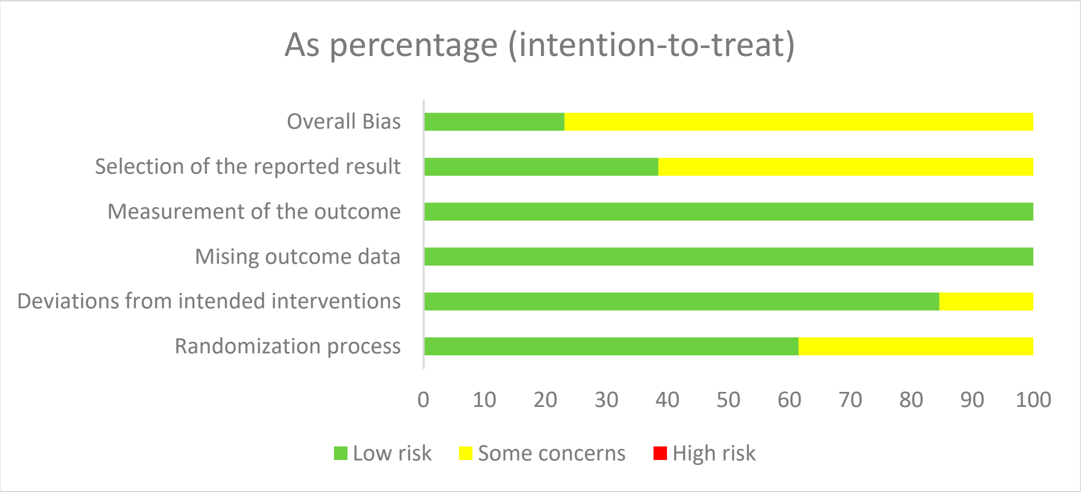

Sup.fig.2: Bias assessment as percentage, after evaluating the studies with RoB-2 tool

	Randomization process	Deviations from intended interventions	Mising outcome data	Measurement of the outcome	Selection of the reported result	Overall Bias
Assignment to intervention (the 'intention-to-treat' effect)						
Total number of study = 13						
Low risk	61,5	84,6	100	100	38,5	23,1
Some concerns	38,5	15,4	0	0	61,5	76,9
High risk	0	0	0	0	0	0

Sup.fig.3: bias assessment as percentage, after evaluating the studies with RoB-2 tool

FOREST AND FUNNEL PLOTS

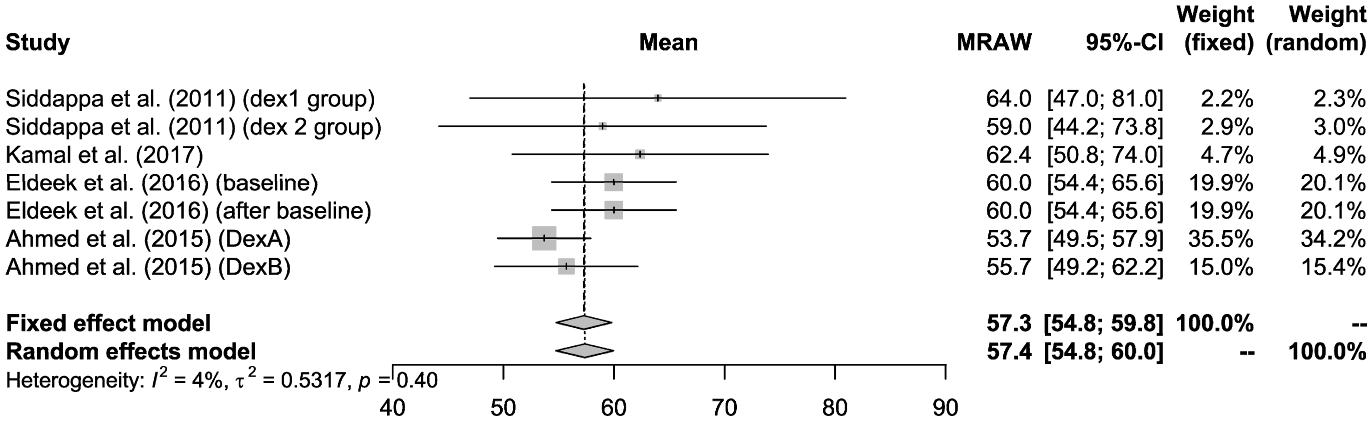

Sup.fig.4: Forest plot of participants' age (months)

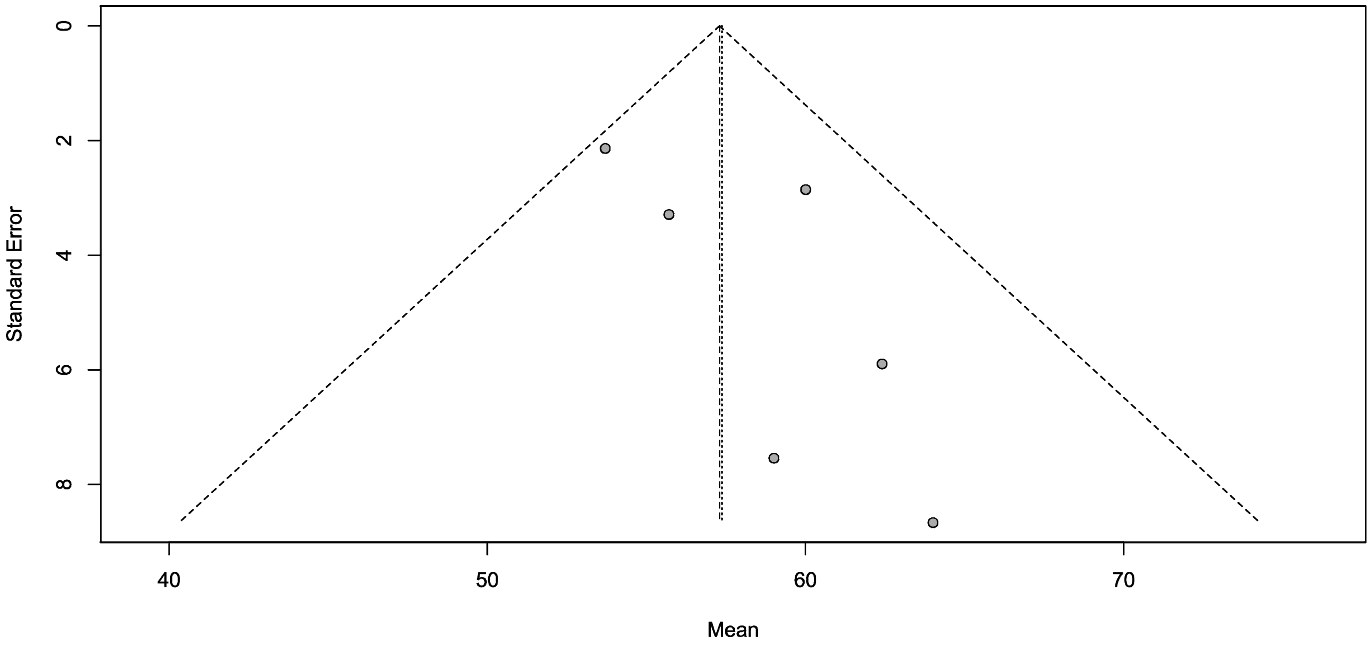

Sup.fig.5: Funnel plot of participants' age (months)

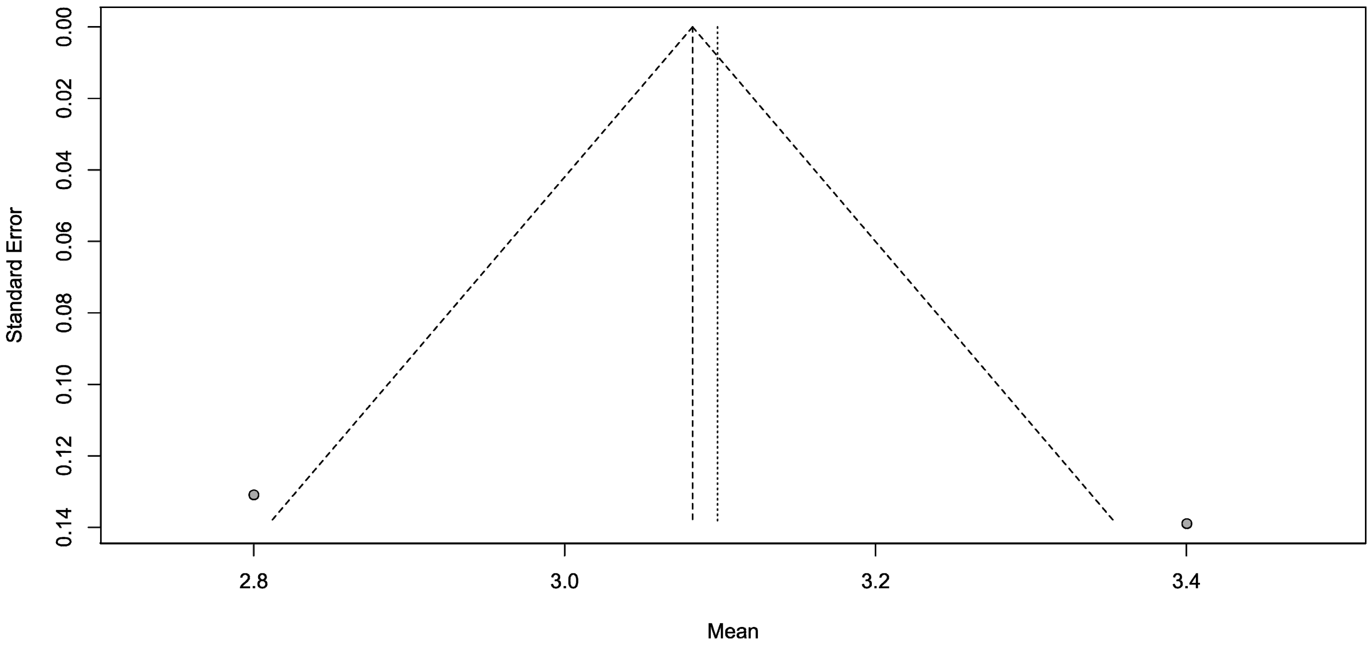

Sup.fig.6: Funnel plot about the induction dose of DEX

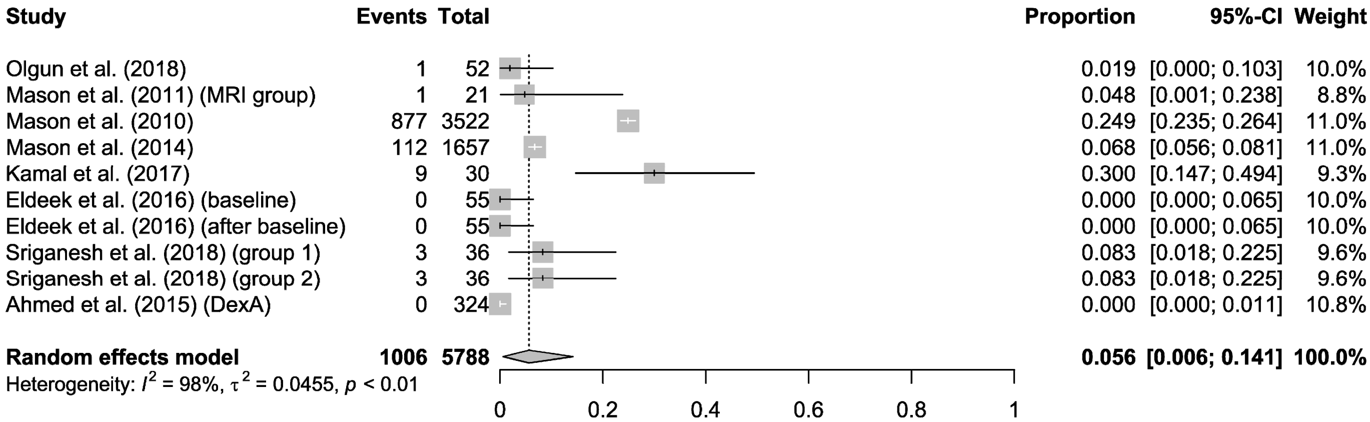

Sup.fig.7: Forest plot about the need of additional dose of DEX

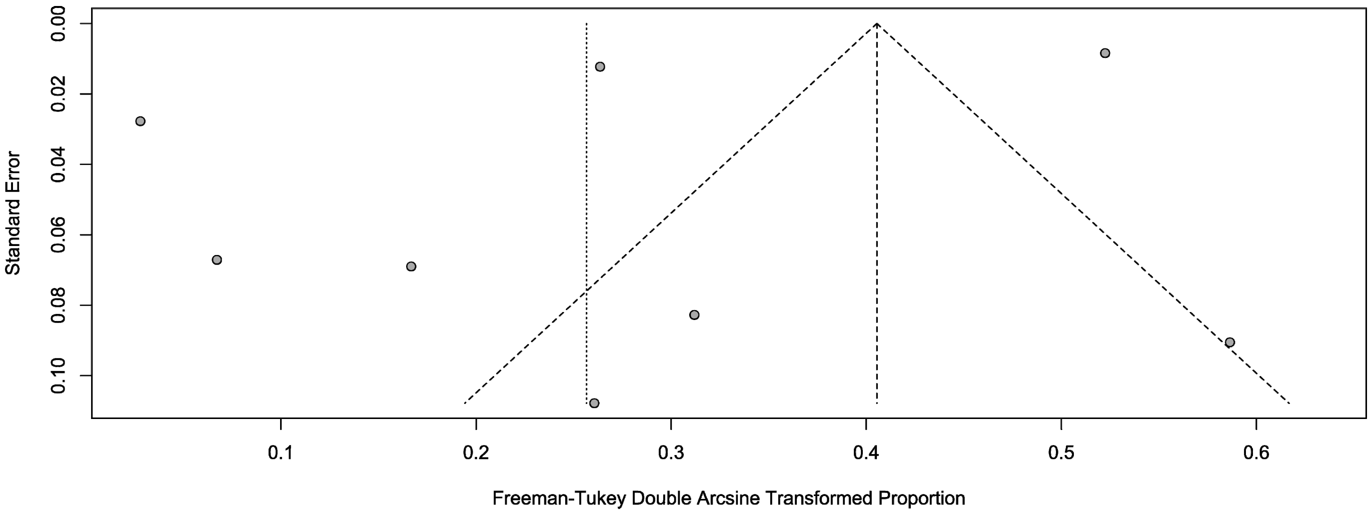

Sup.fig.8: Funnel plot about additional doses of DEX

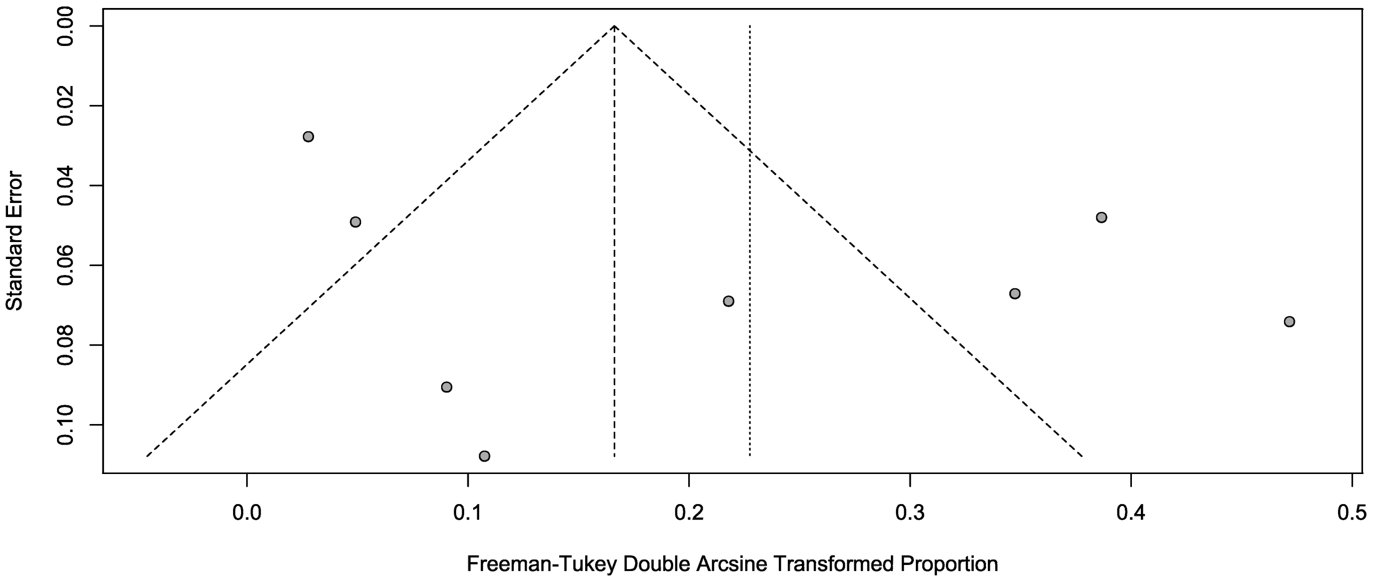

Sup.fig.9: Funnel plot about DEX's failure

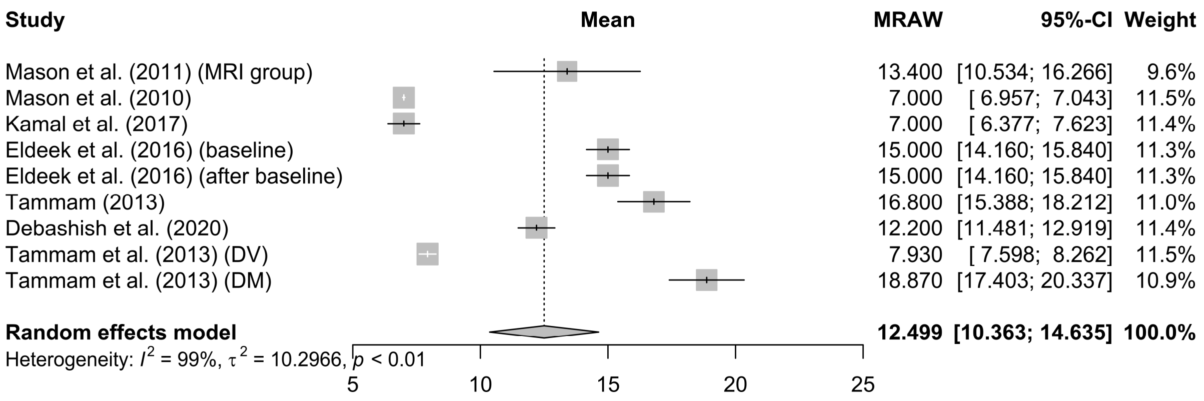

Sup.fig.10: Forest plot of onset time

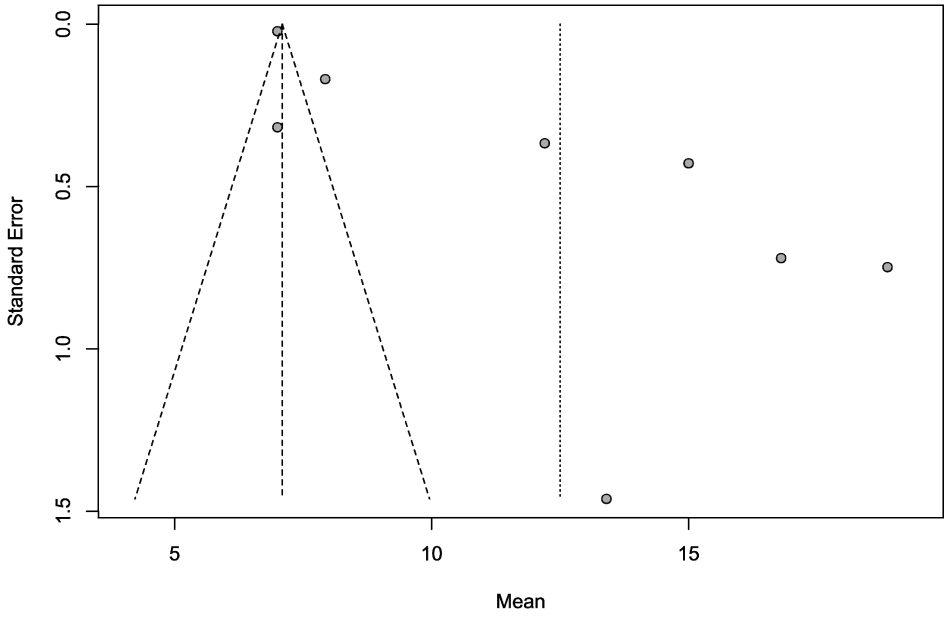

Sup.fig.11: Funnel plot of onset time.

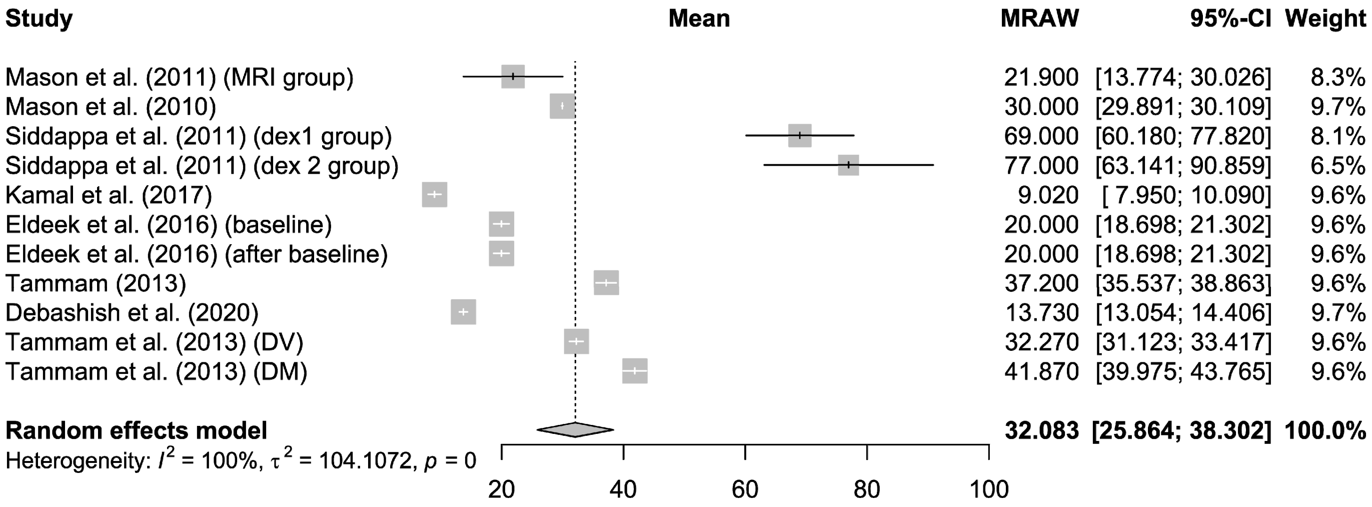

Sup.fig.12: Forest plot for the recovery time

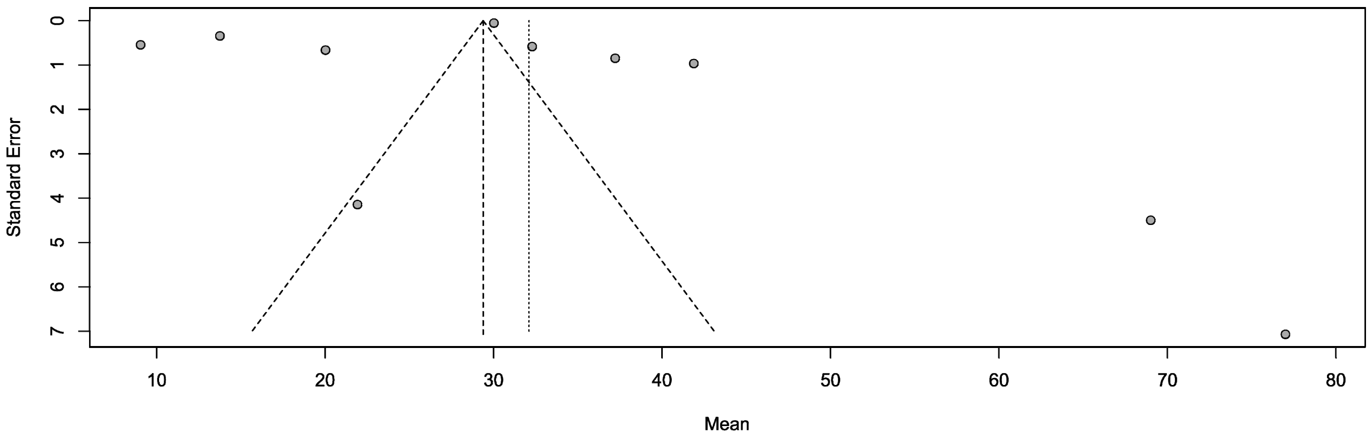

Sup.fig.13: Funnel plot about recovery time

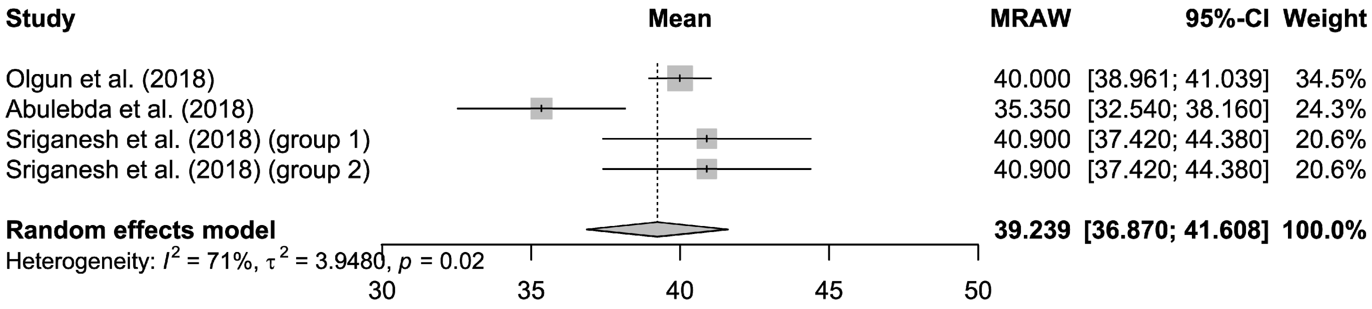

Sup.fig.14: Forest plot of total time

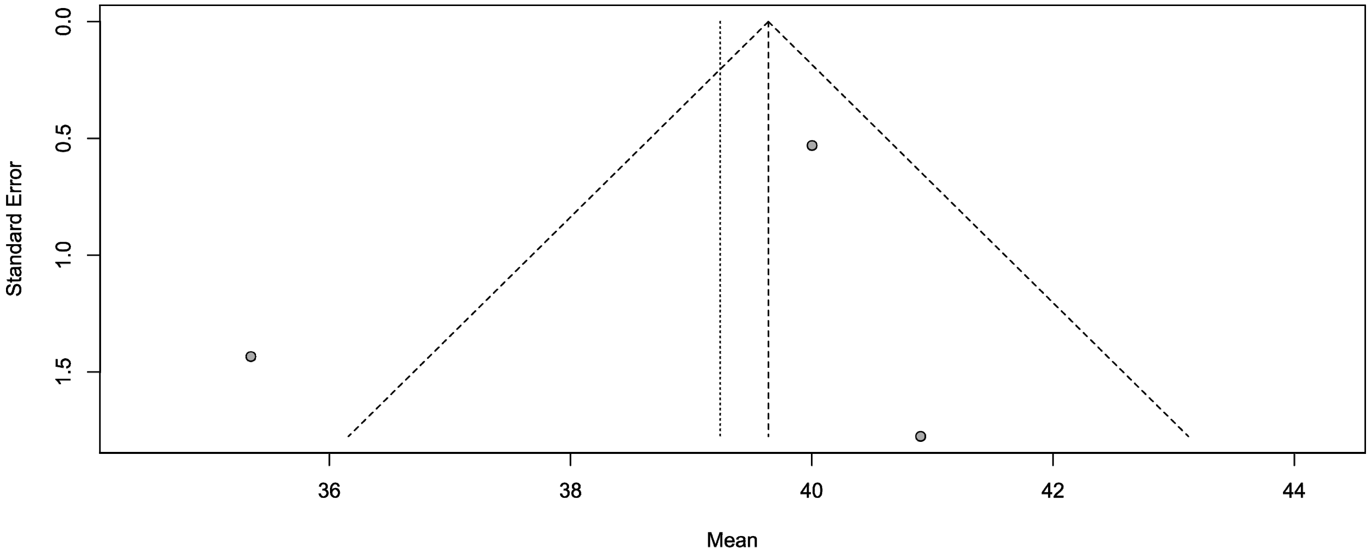

Sup.fig.15: Funnel plot of total time

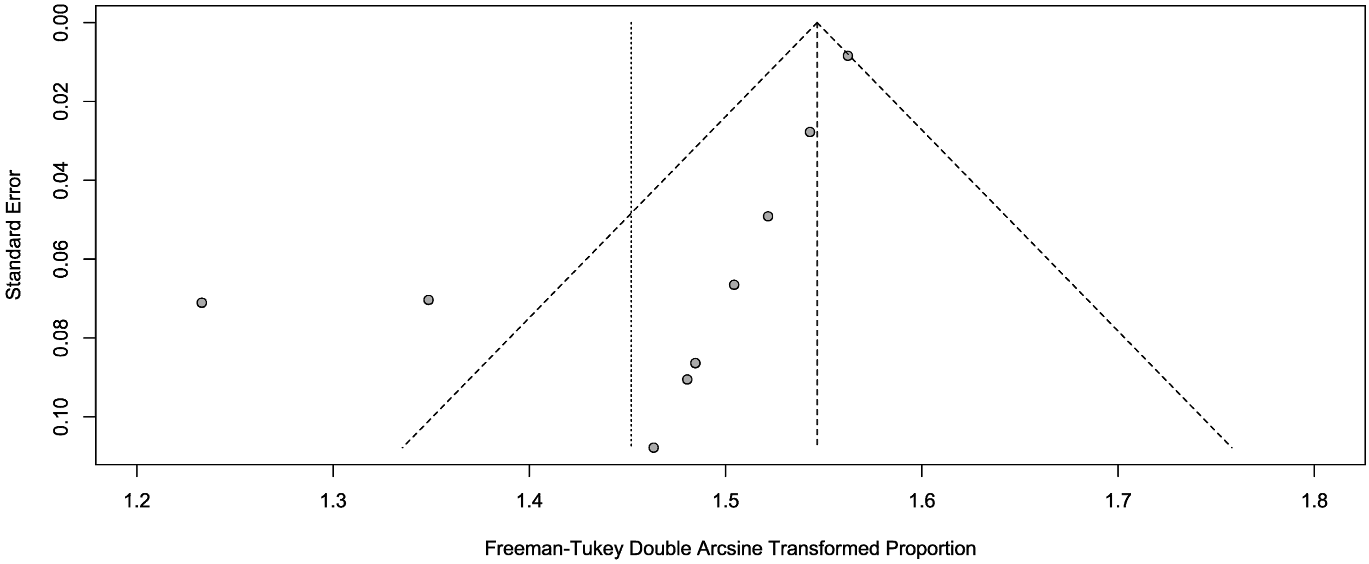

sup.fig.16: Funnel plot of method’s efficiency

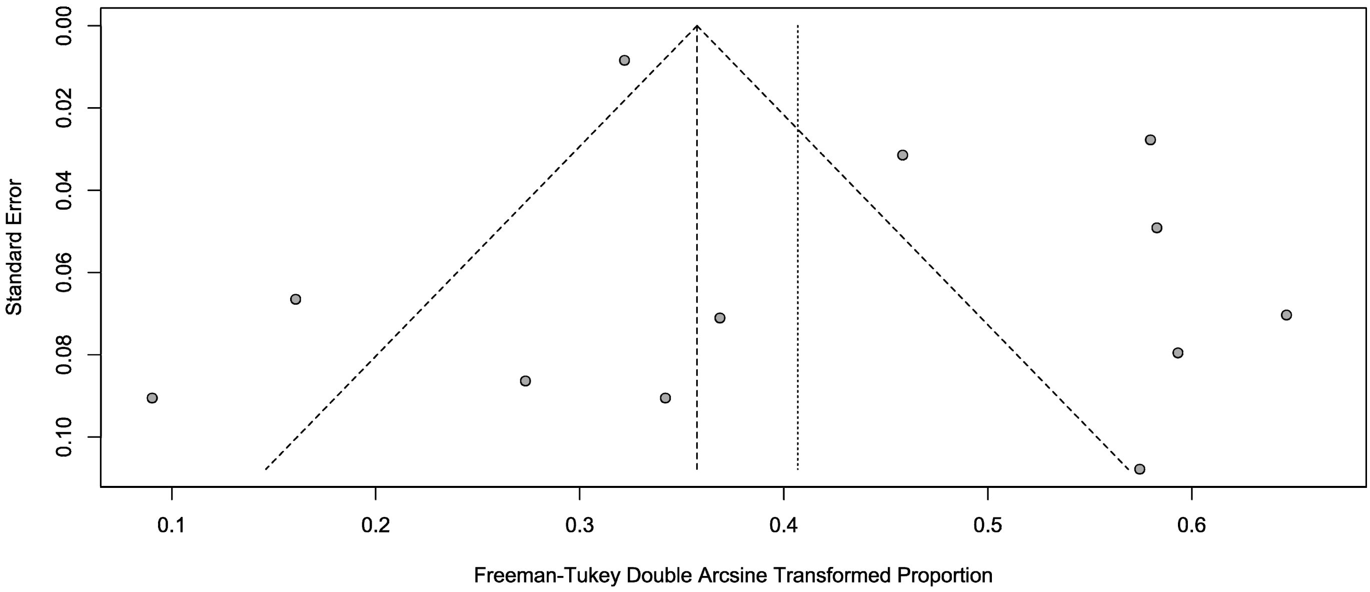

sup.fig.17: Funnel plot of method's safety

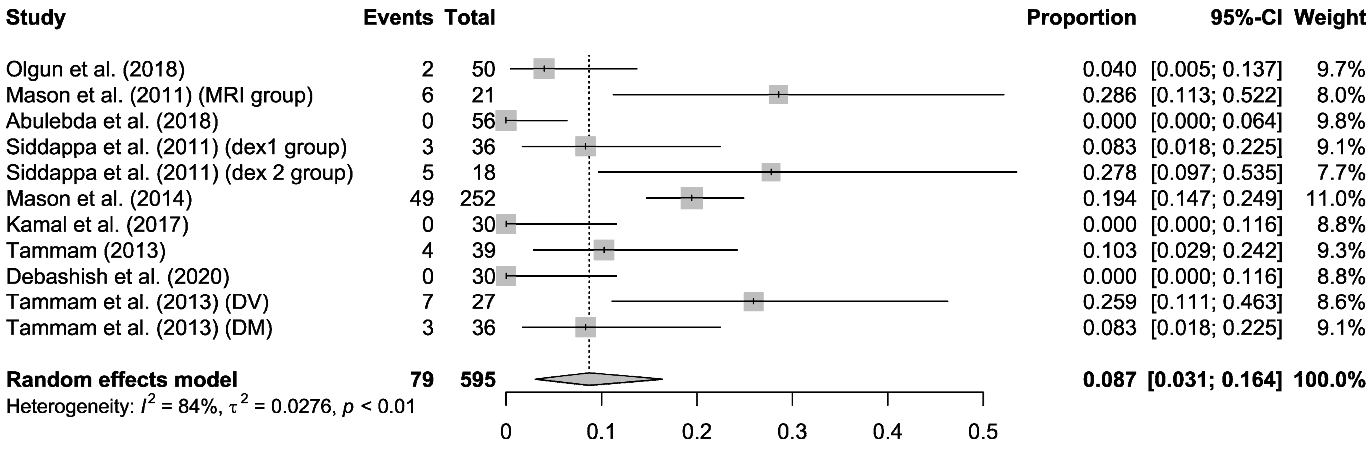

suppl.fig.18: Forest plot of hypotension

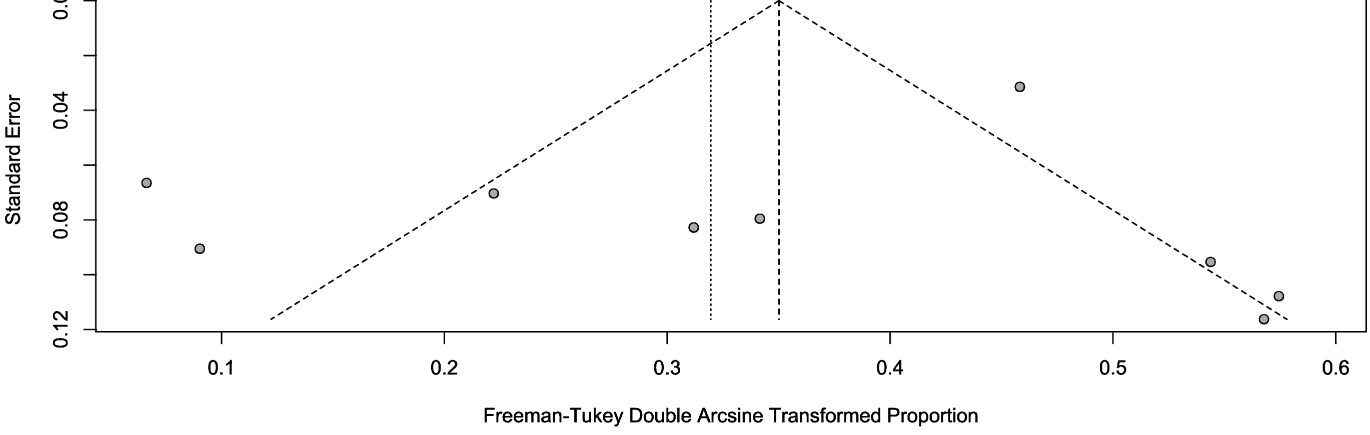

sup.fig.19: Funnel plot for hypotension.

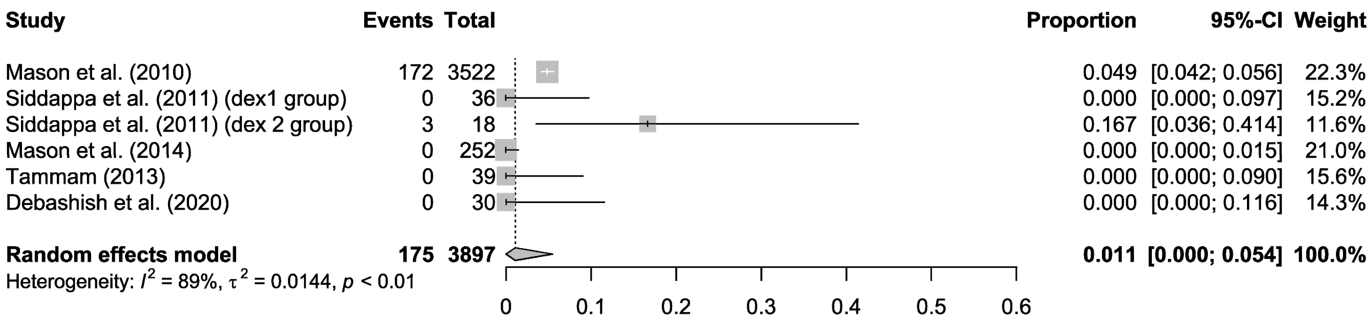

suppl.fig.20: Forest plot of hypertension

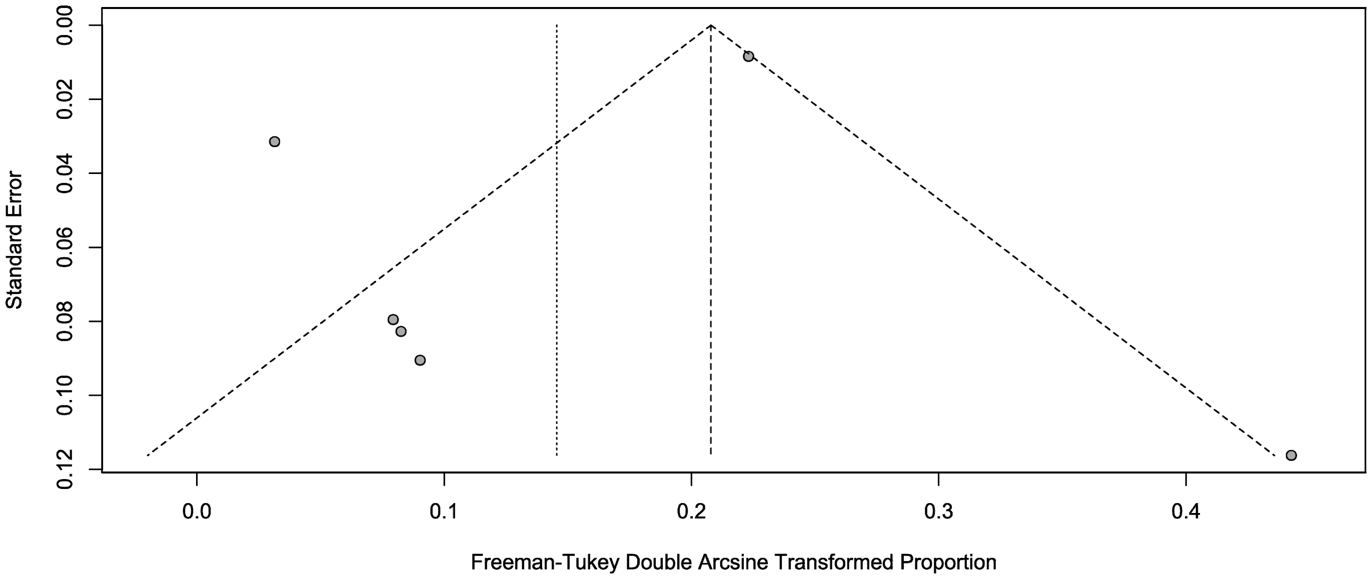

sup.fig.21: Funnel plot for hypertension

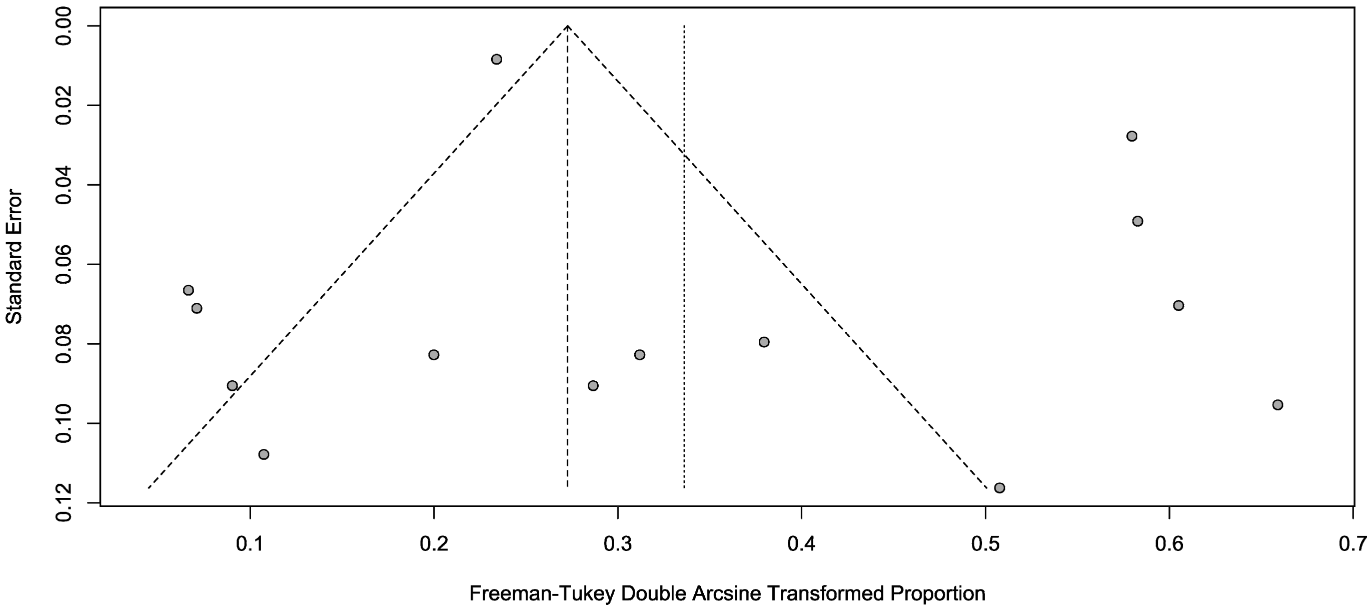

sup.fig.22: Funnel plot for bradycardia

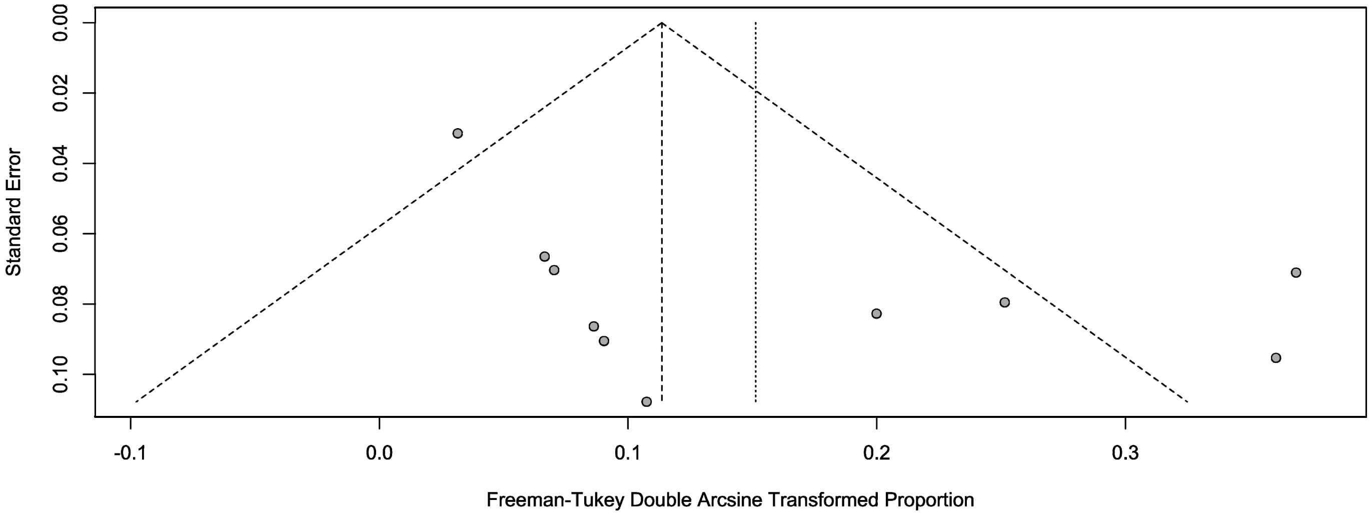

sup.fig.23: Funnel plot for desaturation
